# Oncology research in the kingdom of Bahrain: a four-decade bibliometric evolution

**DOI:** 10.3389/fonc.2026.1829889

**Published:** 2026-06-11

**Authors:** Randah R. Hamadeh, Enjy Khedr, Haitham Jahrami

**Affiliations:** 1 College of Medicine and Health Sciences, Arabian Gulf University, Manama, Bahrain; 2 Benha University, Benha, Egypt; 3 Government Hospitals, Manama, Bahrain

**Keywords:** bibliometric, cancer research, citation analysis, oncology, research productivity, sarcoma

## Abstract

**Introduction:**

Bahrain reports the highest age-standardized cancer incidence among Gulf Cooperation Council (GCC) countries, but its oncology research output has not been comprehensively mapped. We profiled four decades of Bahrain-linked cancer publications to describe growth, contributors, collaboration, impact, and gaps.

**Methods:**

We searched Scopus and PubMed from inception to December 2024 using cancer-related terms combined with “Bahrain”. We included articles, reviews, editorials, letters, conference papers, and book chapters. After deduplication, screening, and manual supplementation, 502 publications were analyzed by year, document type, journal quartile, SCImago Journal Rank (SJR), Bahraini authorship and first-author affiliation, geographic scope, study design, citations, and cancer site grouped using the ICD-O (3rd edition).

**Results:**

The first indexed paper appeared in 1981. Output was low until 2010, then increased sharply; 77.7% of publications appeared from 2011 to 2024, peaking at 66 papers in 2024. Articles accounted for 69.3% of the outputs, and reviews accounted for 21.7%, with greater diversity after 2010. The Bahrain Medical Bulletin was the most common outlet (20.2%). Salmaniya Medical Complex led in first-author output, followed by Arabian Gulf University and King Hamad University Hospital. Bahraini first authorship was observed in 66.7% of papers, and most studies were conducted exclusively in Bahrain (65.3%), although the proportion of global collaborations increased to 17.0% from 2021 to 2024. Descriptive designs predominated (57%, including 25.5% case reports), whereas analytic studies were uncommon (4.3%). Breast cancer was the most studied site-specific cancer (23.3%), followed by digestive organ cancers (11.4%); 27.1% of the papers addressed “unspecified” cancer. Respiratory and intrathoracic cancers remain persistently underrepresented despite a high mortality burden. Journal quality improved after 2010, reflected by increasing Q1/Q2 representation and higher SJR distributions. The citation peaks from 2014 to 2018 were driven by highly cited multinational collaborative publications.

**Conclusions:**

Bahrain’s oncology research output has increased more than 30-fold over four decades, with expanding institutional participation and increasing international collaboration. Persistent reliance on descriptive designs and gaps in high-burden cancers—especially lung cancer—highlights priorities for capacity building, targeted funding, and a nationally aligned oncology research agenda for policy action.

## 1 Introduction

Cancer was the second leading cause of death globally in 2023 after cardiovascular diseases. Over the period of 1990-2023, the number of risk-attributable cancer deaths increased by 72·3% (57·1--86·8), of which more than two-fifths were attributable to established risk factors, mostly shared with other noncommunicable diseases (NCDs), including tobacco smoking, alcohol consumption, obesity, and physical inactivity. Overall, the number of cancer deaths worldwide has increased by 74·3% (62·2-86·2). However, the probability of dying due to cancer between the ages of 30 and 70 years was forecasted to decrease by a relative amount of 6·5% (3.2-10.3) between 2015 and 2030 (1). These projections highlight the urgent global public health challenges associated with the increasing cancer burden in the twenty-first century (2). Implementing primary prevention strategies to reduce exposure to these modifiable risk factors provides broad public health benefits that extend beyond reductions in cancer burden (2). By 2022, the United States experienced a 34% overall reduction in cancer mortality since 1991, which was attributable to smoking cessation, improved early tumor detection, and advances in treatment, especially in immunotherapy and targeted therapy. However, progress in cancer prevention has lagged as the incidence rates for six of the top ten cancers continue to rise, and the cancer burden is shifting from men to women and from older to younger populations (3). In 2023, breast cancer had the highest incidence among all cancer types, followed by cancers of the trachea, bronchus, and lung, as well as colorectal, prostate, and stomach cancers globally. Tracheal, bronchial, and lung cancers accounted for the greatest number of cancer-related deaths, followed by colorectal, stomach, breast, and esophageal cancers (2).

Cancer constitutes an increasing public health concern in the Gulf Cooperation Council (GCC) countries. In 2020, approximately 42,475 new cancer cases and 19,895 cancer-related deaths were reported across the region. The age-standardized incidence and mortality rates were 96.5 and 52.3 per 100,000 population, respectively (4). Bahrain had the highest age-standardized cancer incidence rate among GCC countries in 2022 (5).The five most common cancers in Bahrain in both sexes in 2022 were breast cancer (23.4%), colorectal cancer (11.4%), non-Hodgkin’s lymphoma (NHL) (6.6%), lung cancer (6.4%), and prostate cancer (5.4%), whereas the five leading causes of cancer mortality were breast (15.8%), lung (12.7%), colorectal (10.7%), leukemia (5.6%), and stomach cancers (5.4%) (5). In the Bahrain Ministry of Health cancer incidence and mortality reports for 1998–2016 and 2009-2018, the top four leading cancers in both sexes were similar (breast cancer, colorectal cancer, lung cancer, and NHL), whereas the 5th was bladder cancer and leukemia in the earlier period and bladder cancer in the latter (6, 7).

The World Health Organization (WHO) has stressed the importance of research productivity as a foundation for evidence-based practice. The WHO stated that “Research and the evidence that research yields are critical elements for improving global health and health equity, as well as economic development” (8). In oncology, adherence to current evidence-based guidelines and the foundational research that underpins them is critical for providing optimal cancer care. Furthermore, continued improvements in cancer survival rates require significant contributions from basic science, translational research, and outcome-based investigations (9).

Bibliometric analysis has become a standardized and widely adopted methodology for systematically characterizing, quantifying, and assessing scientific contributions, including journal publications, within specific disciplines (10). Research councils and institutes commonly employ bibliometric data analysis to gain an external perspective on a research field (11). This approach also provides valuable information to stakeholders, such as policymakers, researchers, healthcare practitioners, and patients, regarding the nature, quantity, and impact of publications in relevant fields over defined time periods (10). Bibliometric studies are also necessary to analyze published literature and identify current research trends and gaps (12).

Although cancer research productivity in Arab countries has shown a steady upward trend, it remains relatively low compared with that in other regions of the world. Political unrest and conflict in several Arab nations have led to a de-prioritization of research funding. Furthermore, some countries experience significant brain drain among researchers, and the prevailing research environment is often unsupportive. These factors collectively contribute to the lower output of cancer research in Arab nations (13, 14). Breast cancer is the most researched cancer and is prioritized over other prominent malignancies, followed by colorectal and liver cancer in the Arab region (13, 14). Bahrain had the highest proportion of articles published on breast cancer research (44.2%) among Arab countries (14).

Over the past decade, GCC countries have experienced consistent growth in cancer-related publications, patents, and innovations. However, challenges persist regarding the sustainability and scale of research funding. Although more high-quality cancer research is necessary in GCC countries to generate evidence on cancer profiles and determinants and to better understand cancer status in the Gulf region to inform cancer control, research expenditures remain low (15). In 2023, global research and development expenditures (GERD) averaged 1.92% of gross domestic product (GDP), yet GCC countries invested below this average. The United Arab Emirates reported the highest GERD at 1.49% of GDP, followed by Qatar (0.68%), Saudi Arabia (0.64%), Oman (0.39%), Bahrain (0.19%), and Kuwait (0.09%) (16).

Bahrain has experienced a rising cancer burden and currently has the highest age-standardized cancer incidence rate for both sexes among GCC countries (17). A significant gap in the literature is the absence of a comprehensive, long-term bibliometric assessment of cancer research in Bahrain. Among the 1,773 cancer publications identified from seven Arab countries (Bahrain, Kuwait, Iraq, Lebanon, Morocco, Palestine, and Sudan) between 2000 and 2013, Bahrain contributed 2.1% (13). The evolution of cancer research output in relation to changing disease burdens, health system priorities, and regional research trends remains unclear. This study aims to quantitatively assess cancer-related research output in Bahrain from the start of indexing in Scopus and PubMed to 2024 and to identify research gaps. The study objectives were to describe the temporal patterns of cancer research, identify the most and least commonly researched cancers, compare the cancer studies conducted in Bahrain to those conducted in countries where Bahrain was part of the study, compute the percentage of publications with the first author from Bahrain, and determine the most common study designs used.

## 2 Materials and methods

A comprehensive bibliometric search was conducted to identify all publications on cancer in Bahrain up to 31 December 2024. The first research strategy employed Scopus, which uses a combination of keywords (malignancy) OR (cancer) OR (neoplasm) OR (carcinoma) OR (sarcoma) AND Bahrain) as the affiliation country. This search yielded 833 documents. The second Scopus search was conducted using the following keywords: (malignancy) OR (cancer) OR (neoplasm) OR (carcinoma) OR (sarcoma), AND Bahrain, in any field of the document. This search yielded 2,355 documents. The third search used PubMed and was conducted with the following keywords: (malignancy) OR (cancer) OR (neoplasm) OR (carcinoma) OR (sarcoma), AND Bahrain, in any field of the document. This search yielded 447 documents.

Eligible document types included articles, editorials, letters, notes, short surveys, conference papers, books, book chapters, and reviews. Articles in press or errata were excluded. All languages were included in the study. The resulting dataset was exported as a CSV file and subsequently imported into Excel, capturing all authors’ full names, titles, publication years, source titles, volumes, issues, DOIs, links, affiliations, author affiliations, the languages of the original documents, and document types. The three files were merged, resulting in 3635 documents. Duplicates (1231 documents) were deleted via the Excel duplicate identifier. Withdrawn (1 document) and retracted (3 documents) were excluded, resulting in a final count of 2400 documents. Documents that were not about Bahrain or not related to malignancy (1406 documents) or reviews, books, and book chapters that did not include Bahrain (427 documents), documents about benign tumors (61 documents), and medical quizzes, medical news, or multiple-choice questions (18 documents) were excluded from the study. Only publications meeting the final inclusion criteria were selected. Therefore, the final count reached 488 documents. Finally, a manual search was performed to identify all the documents related to cancer in Bahrain. This identified 14 additional documents, which were added to the main file, resulting in 502 documents identified and analyzed.

**Figure 1:**
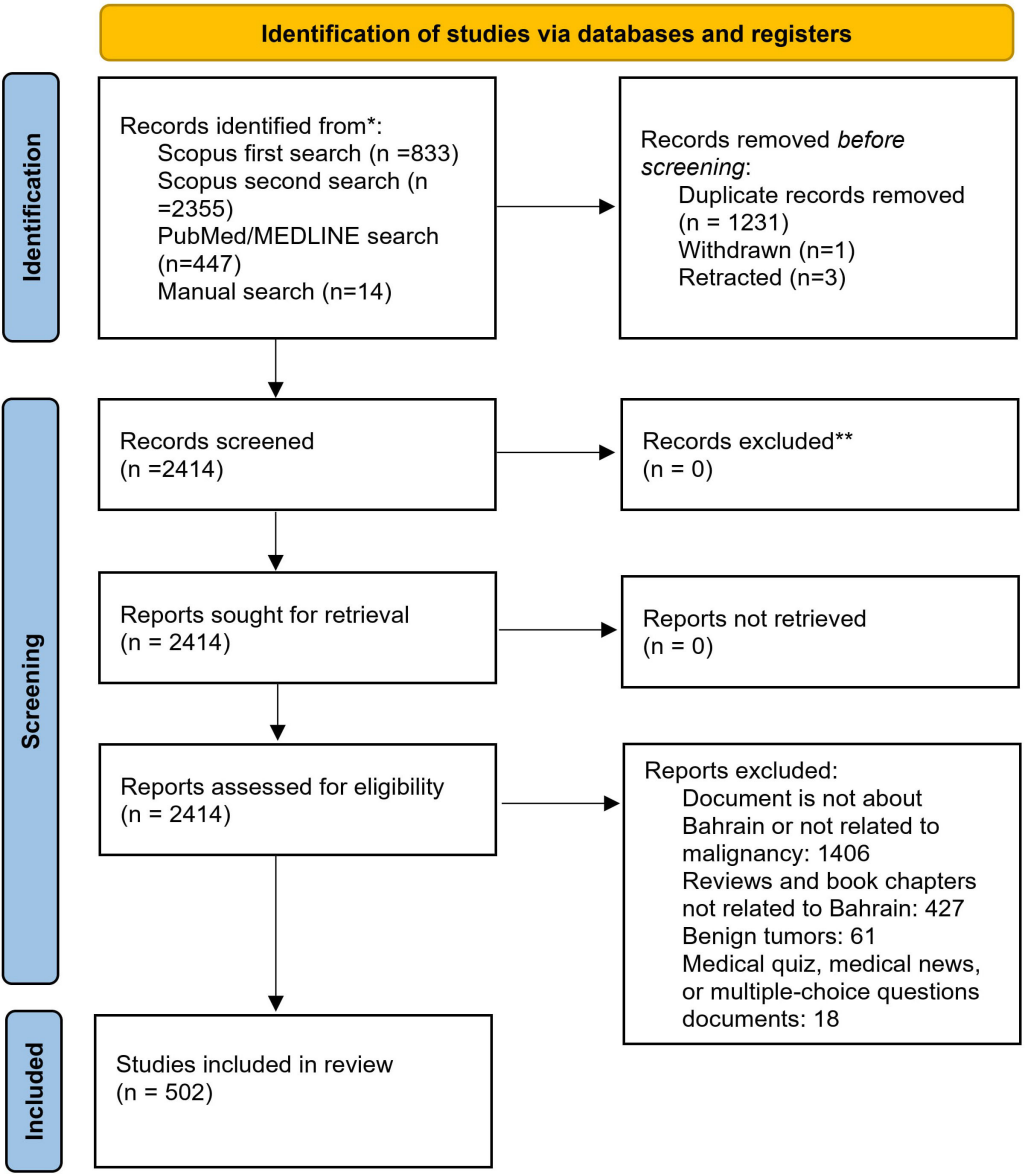
Identification of studies via databases and registries. Flow diagram detailing the systematic search and selection of publications. Searches in Scopus (two strategies) and PubMed initially retrieved 3635 records. After removing duplicates (n=1231), excluding irrelevant/retracted/withdrawn items (n=1916), and adding 14 manually identified records, 502 documents were included in the bibliometric analysis. Search date: 24 September 2025; articles in press excluded.

To provide a comprehensive characterization of the research landscape, several additional bibliometric parameters were extracted and analyzed. The institutional affiliation of the first author was recorded for all publications authored by individuals from Bahrain and categorized by institution type (clinical vs. academic). The geographic scope of each publication was classified into five categories: Bahrain only, GCC, Arab countries, Middle East and North Africa/Eastern Mediterranean Region (MENA/EMR), and global. If the geographic scope was not specified in the document, reliance was placed on information from the ethical approval, the funding agency, the author contributions, the keywords, and a search for Bahrain within the document. The search strategy used in this study is described in **Figure 1**. Journal SCImago Journal Ranking (SJR) values were retrieved from the Journal Citation Reports for each document’s publication year and analyzed by decade using violin plots overlaid with boxplots on a log10 scale to account for skewed distributions. The composition of document types was tracked annually to examine temporal shifts in the mix of articles, reviews, book chapters, editorials, letters, and notes. Citation analysis was conducted by calculating total and mean citations per paper per year, identifying the ten most-cited publications. A cancer site-by-study cross-tabulation design was generated as a heatmap to reveal methodological gaps at the disease-site level. The publication trends for the five most-researched cancer site groups were plotted annually using LOESS smoothing to visualize site-specific trajectories over time.

Cancers were grouped according to the International Classification of Diseases for Oncology (ICD-O), third edition (18). Documents that focused on cancer in general without a specific cancer site were grouped as “unspecific cancer.” To make a rough comparison of Bahrain-affiliated oncology and cancer-related research output with international trends, we used global PubMed-indexed publications in cancer and oncology as a benchmark for the broader biomedical literature landscape.

The normality of the distributions of the variables analyzed was assessed via the Kolmogorov–Smirnov test. All the numerical data were nonnormally distributed. The collected data are summarized as the median and interquartile range (IQR), or the mean and standard deviation (SD), for quantitative data, and as counts and percentages for qualitative data. For categorical variables, comparisons between study groups were performed using Fisher’s exact test. The Kruskal–Wallis test was used to compare more than two groups of nonnormally distributed quantitative variables. All tests were two-sided. The accepted level of significance in this work was p <0.05.

All additional bibliometric analyses and visualizations were performed in R (R version 4.5.3 (Reassured Reassurer) was released on 2026-03-11; R Foundation for Statistical Computing, Vienna, Austria) via the following packages: readxl for data import, dplyr and tidyr for data manipulation, stringr for text processing, ggplot2 as the core visualization engine, ggtext for text rendering, scales for axis formatting, forcats for factor handling, patchwork for figure composition, RColorBrewer and viridis for color palettes, lubridate for date handling, treemapify for treemap visualization, and ggwordcloud for word cloud generation.

## 3 Results

### 3.1 Temporal trends of publications

**Figure 2:**
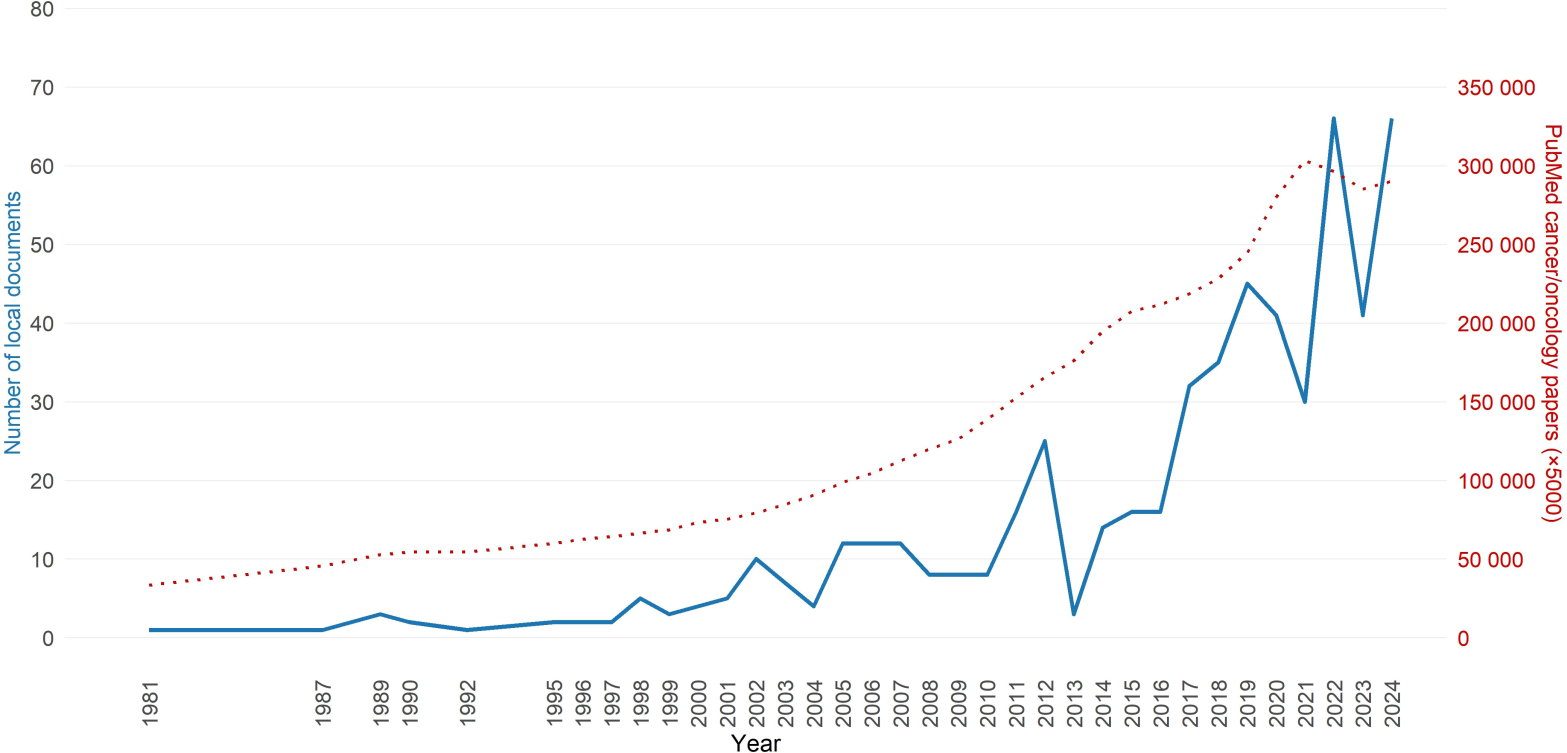
Publication trends over time. Annual number of oncology- and cancer-related publications with Bahrain affiliation or focus (solid blue line, left y-axis) from 1981 to 2024, shown alongside approximate global annual PubMed-indexed cancer/oncology publications (dashed red line, right y-axis, scaled ×5000).

A total of 502 cancer-related publications have been produced in Bahrain since it first appeared in 1981. Over the past four decades, the trends and growth of oncology research output in Bahrain have changed substantially. The publication output in Bahrain remained consistently low and stable for the first three decades of the study period, with very few documents produced annually until the early 2000s. The average annual output increased from fewer than one paper per year in the 1980s to more than 45 per year in the current decade (**Figure 2**, **Table 1**). The dramatic acceleration in productivity began after 2010; 77.9% of all identified research was published between 2011 and 2024. The most productive years were 2024, 2022, and 2023, which accounted for 13.1%, 9.0%, and 8.2% of the total output, respectively, with a total of 30.3% (**Figure 2**). While the total output for 2011–2020 was the highest (41.4%), the year-adjusted productivity rate in the 2021–2024 period more than doubled (45.5%) compared with 20.8% during 2011–2020, with 182 publications in the last four-year period (**Table 1**). In contrast, global PubMed publications in cancer and oncology presented a steady, progressive upward trajectory over the same period. This pattern reflects sustained international research expansion, increasing biomedical knowledge, and consistent annual growth in the overall literature, without the abrupt surges observed in Bahrain’s data (**Figure 2**).

**Table 1:** Comparison of authorship, research site, study design, quartile, SJR, cancer type, and time periods.

Variables		1981–1990 (n=7, 1.4%)	1991–2000 (n=19, 3.8%)	2001–2010 (n=86, 17.1%)	2011–2020 (n=208, 41.4%)	2021–2024 (n=182, 36.3%)	p-value
First author from Bahrain*		6 (85.7)	19 (100.0)	71 (82.6)	137 (65.9)	102 (56.0)	<0.001
At least one Bahraini author*		7 (100.0)	19 (100.0)	75 (87.2)	163 (78.4)	136 (74.7)	0.014
Research Site*	Bahrain	7 (100.0)	17 (89.5)	67 (77.9)	133 (63.9)	104 (57.1)	0.003
	Global	0 (0.0)	0 (0.0)	3 (3.5)	29 (13.9)	31 (17.0)	
	GCC/Arab/MENA	0 (0.0)	2 (10.5)	16 (18.6)	46 (22.1)	47 (25.8)	
Study design*	Descriptive	2 (28.6)	13 (68.4)	48 (55.8)	117 (56.3)	97 (58.4)	0.272
	Analytic	1 (14.3)	1 (5.3)	3 (3.5)	4 (1.9)	12 (7.2)	
	Reviews	2 (28.6)	4 (21.1)	23 (26.7)	51 (24.5)	30 (18.1)	
	Others	2 (28.6)	1 (5.3)	12 (14.0)	36 (17.3)	27 (16.3)	
Quartile (n=420)*	Q1	0 (0.0)	1 (14.3)	2 (2.4)	55 (27.8)	43 (32.3)	<0.001
	Q2	0 (0.0)	2 (28.6)	9 (11.0)	30 (15.2)	26 (19.5)	
	Q3	0 (0.0)	3 (42.9)	49 (59.8)	43 (21.7)	31 (23.3)	
	Q4	0 (0.0)	1 (14.3)	22 (26.8)	70 (35.4)	33 (24.8)	
SJR (n=420)**		0.0 (0.0-0.0)	0.1 (0.1-0.2)	0.1 (0.1-0.2)	0.4 (0.1-1.0)	0.5 (0.2-1.0)	<0.001
Citations**		2.0 (0.0-22.0)	3.0 (0.0-16.0)	1.5 (0.0-12.0)	9.0 (1.0-40.8)	2.0 (0.0-7.0)	<0.001
Cancer site	Cancer, unspecified	2 (28.6)	5 (26.3)	22 (25.6)	53 (25.5)	54 (29.7)	0.007
	Breast (C50)	0 (0.0)	3 (15.8)	13 (15.1)	59 (28.4)	42 (23.1)	
	Digestive organs (C15-26)	0 (0.0)	1 (5.3)	7 (8.1)	25 (12.0)	24 (13.2)	
	Female genital organs (C51-C58)	1 (14.3)	2 (10.5)	9 (10.5)	18 (8.7)	18 (9.9)	
	Respiratory system and intrathoracic organs (C30-C39)	1 (14.3)	1 (5.3)	11 (12.8)	9 (4.3)	7 (3.8)	
	Urinary tract (C64-C68)	0 (0.0)	1 (5.3)	5 (5.8)	6 (2.9)	12 (6.6)	
	Hematopoietic and reticuloendothelial systems (C42)	1 (14.3)	3 (15.8)	4 (4.7)	5 (2.4)	5 (2.7)	
	Thyroid and other endocrine glands (C73-V75)	0 (0.0)	2 (10.5)	6 (7.0)	7 (3.4)	2 (1.1)	
	Eye, brain, and other parts of the central nervous system (C69-C72)	0 (0.0)	0 (0.0)	1 (1.2)	6 (2.9)	7 (3.8)	
	Lip, oral cavity and pharynx (C00-C14)	1 (14.3)	1 (5.3)	1 (1.2)	2 (1.0)	6 (3.3)	
	Male genital organs (C60-C63)	0 (0.0)	0 (0.0)	2 (2.3)	7 (3.4)	2 (1.1)	
	Skin (C44)	0 (0.0)	0 (0.0)	4 (4.7)	5 (2.4)	1 (0.5)	
	Bones, joints and articular cartilage (C40-C41)	1 (14.3)	0 (0.0)	1 (1.2)	2 (1.0)	2 (1.1)	
	Connective, subcutaneous and other soft tissues (C49)	0 (0.0)	0 (0.0)	0 (0.0)	4 (1.9)	0 (0.0)	

*%, ** median and interquartile range, Fisher exact test, Kruskal–Wallis test.

### 3.2 Document type composition over time

**Figure 3:**
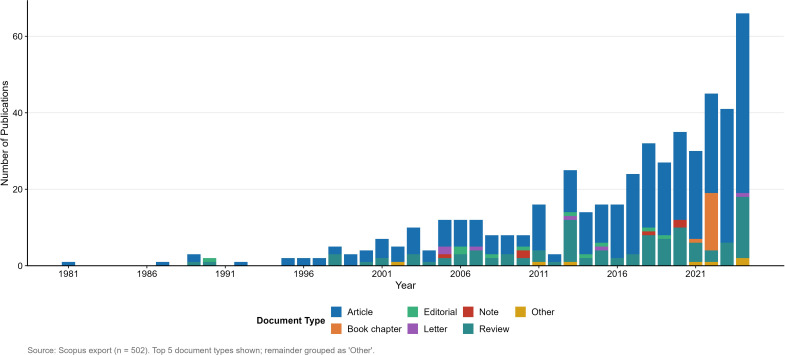
Temporal distribution of publications by document type. Stacked bar chart showing the annual distribution of cancer-related publications with Bahrain affiliation or focus by Scopus document type from 1981 to 2024. The five most common document types are displayed separately—article, book chapter, editorial, letter, note, and review—while the remaining document types are grouped as “Other.”.

Among the 502 publications identified by Scopus during the study period, the majority (349; 69.3%) were classified as articles, and 109 (21.7%) were classified as reviews. There were 16 (3.2%) book chapters and 5 conference papers (**Table 2**). Original articles consistently accounted for the predominant share of publications across all periods, constituting most of the annual output throughout the study period. From approximately 2010 onward, reviews emerged as a significant secondary document type, with increases observed in both absolute and proportional terms. Book chapters were published primarily between 2020 and 2024, coinciding with the release of regional oncology textbooks. Editorials, letters, and notes remained minor components throughout the study period. The overall diversification of document types from 2010 onward signifies the maturation and expansion of Bahrain’s oncology research ecosystem (**Figure 3**).

**Table 2:** Document type and citations.

Document type	No	%
Article	348	69.3
Review	109	21.7
Book chapter	16	3.2
Editorial	10	2.0
Letter	6	1.2
Note	6	1.2
Conference paper	5	1.0
Short Survey	2	0.4
Total	502	100.0
Citations	Median and Interquartile	3.0 (0.0-18.0)

### 3.3 Journal features

**Figure 4:**
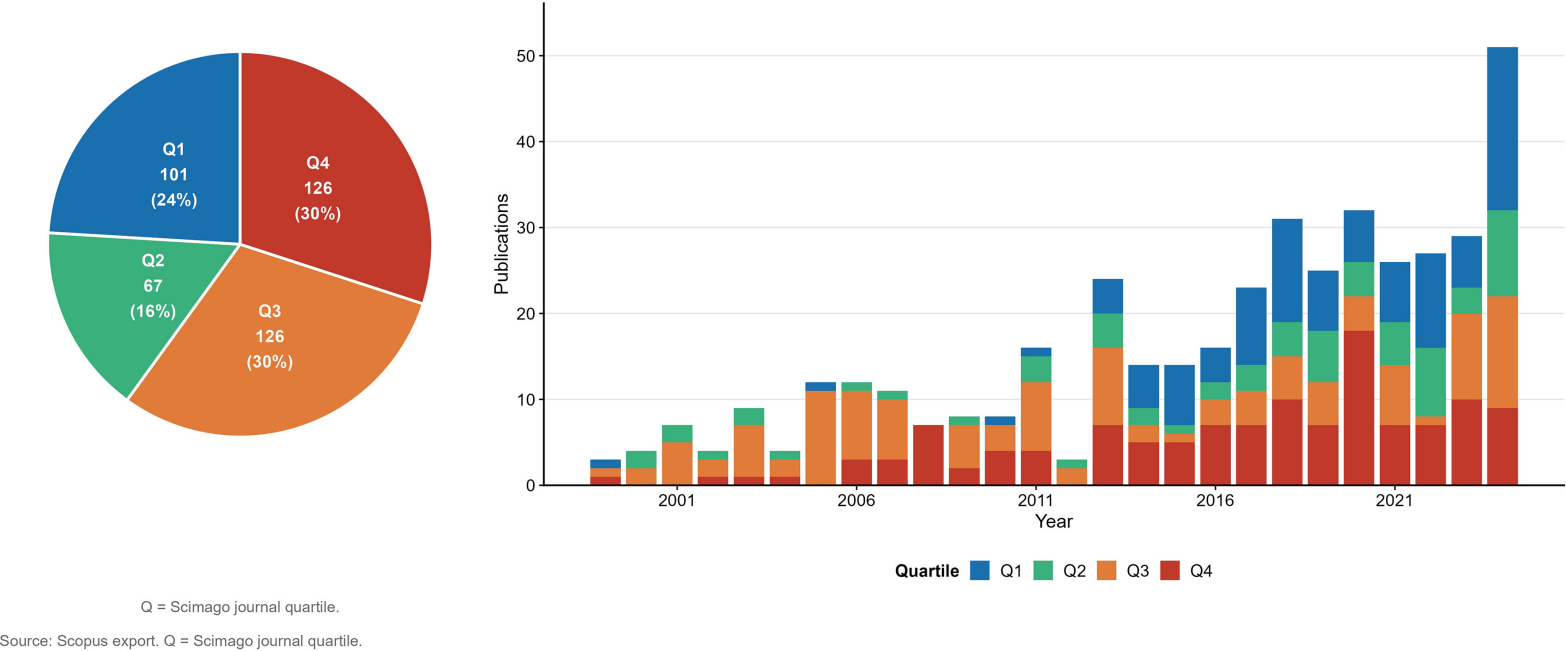
Distribution and temporal pattern of publications by journal quartile. Two-panel figure summarizing oncology- and cancer-related publications with Bahrain affiliation or focus by Scimago journal quartile.

There were 207 journals, all in English. The Bahrain Medical Bulletin was the leading venue, accounting for one-fifth of all journal publications. The journals that had 10 or more publications are shown in **Table 3**. Sixty-six (13.6%) articles had no assigned quartile. The quartiles for the remaining papers are shown in **Figure 4**. Forty percent of the articles that had a quartile assigned were in Q1 and Q2 journals, and the rest (60.0%) were in Q3 and Q4. The mean ± SD and median of the journals were 1.3 ± 3.1 and 0.3 (0.1-0.9), respectively. An increase in the number of Q1 and Q2 journals (p < 0.001) was observed, with Q1 journals reaching almost one-third and Q2 journals almost one-fifth of the total in 2021-2024 (**Table 1**).

**Table 3:** Top journals for Bahraini oncology research with more than 10 publications (n=481).

Source title	Publishing country	Scope	No	%
Bahrain Medical Bulletin	Bahrain	Local	97	20.2
Journal of the Bahrain Medical Society	Bahrain	Local	22	4.6
Saudi Medical Journal	Saudi Arabia	Regional	17	3.5
Cureus	USA	International	15	3.1
The Lancet	United Kingdom	International	15	3.1
Eastern Mediterranean Health Journal	Egypt	Regional	13	2.7
The Gulf Journal of Oncology	Kuwait	Regional	13	2.7

**Figure 5:**
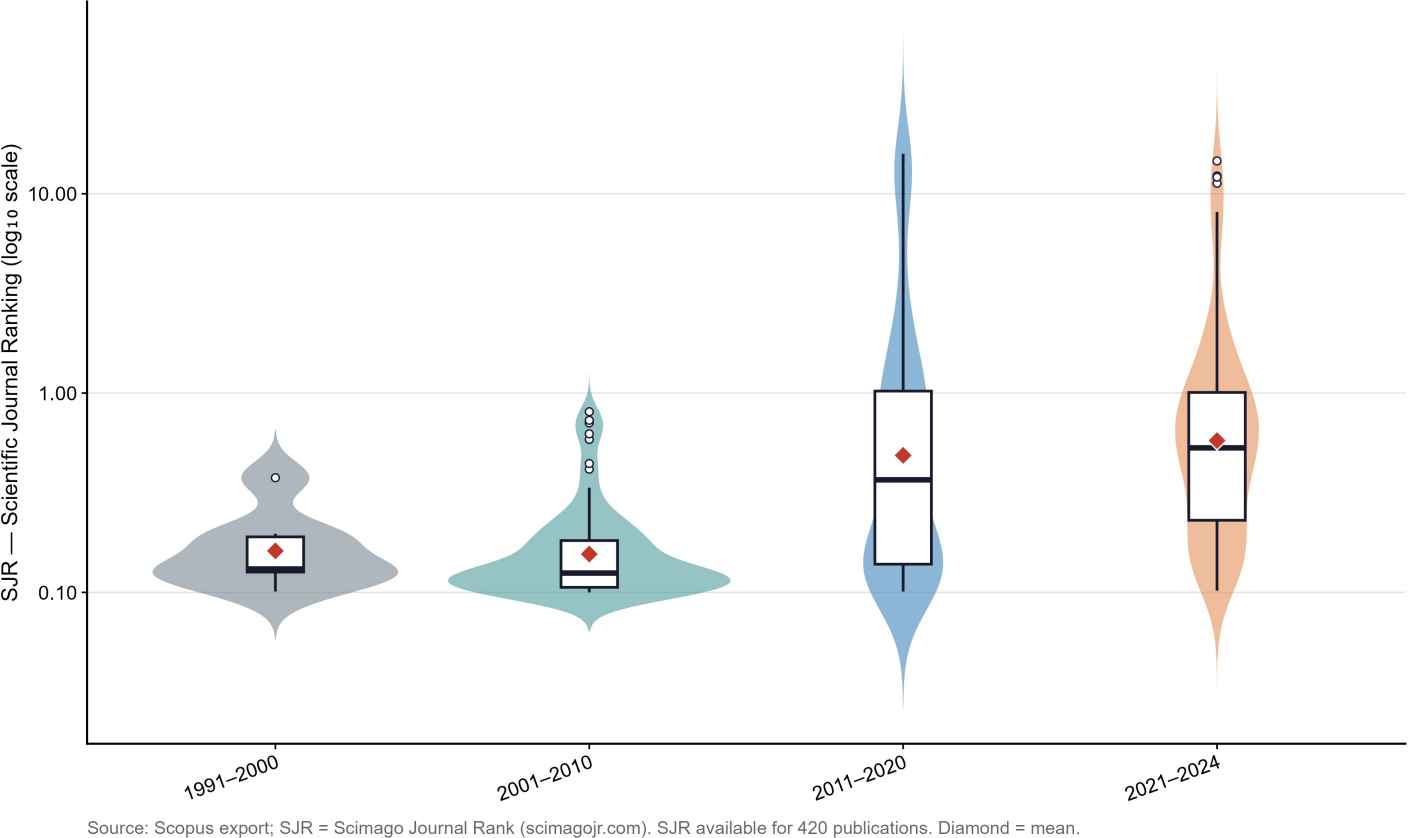
Scientific journal rank distribution by publication period. Violin and box plots showing the distribution of Scientific Journal Rank (SJR) values for oncology- and cancer-related publications with Bahrain affiliation or focus across publication periods. SJR values are displayed on a log10 scale, with violin width indicating the density of publications, embedded box plots showing the median and interquartile range, open circles indicating outliers, and red diamonds representing mean SJR values. SJR data were available for 420 publications.

SJR data were available for 420 out of 502 publications. The distribution of SJRs showed a significant increase when comparing the periods 1990–1999 and 2000–2009, during which the median SJRs remained below 0.2, and the violin plots displayed narrow, low-value distributions for the periods 2010–2019 and 2020–2024. In these latter periods, both the median and mean SJRs increased substantially, and the distribution broadened considerably, indicating a rising proportion of publications in higher-impact international journals. A small number of publications with exceptionally high SJRs (greater than 10) emerged during the 2010–2019 decade, corresponding to the multinational collaborative papers identified in the citation analysis (**Figure 5**, **Table 1**).

### 3.4 Citation analysis

**Figure 6:**
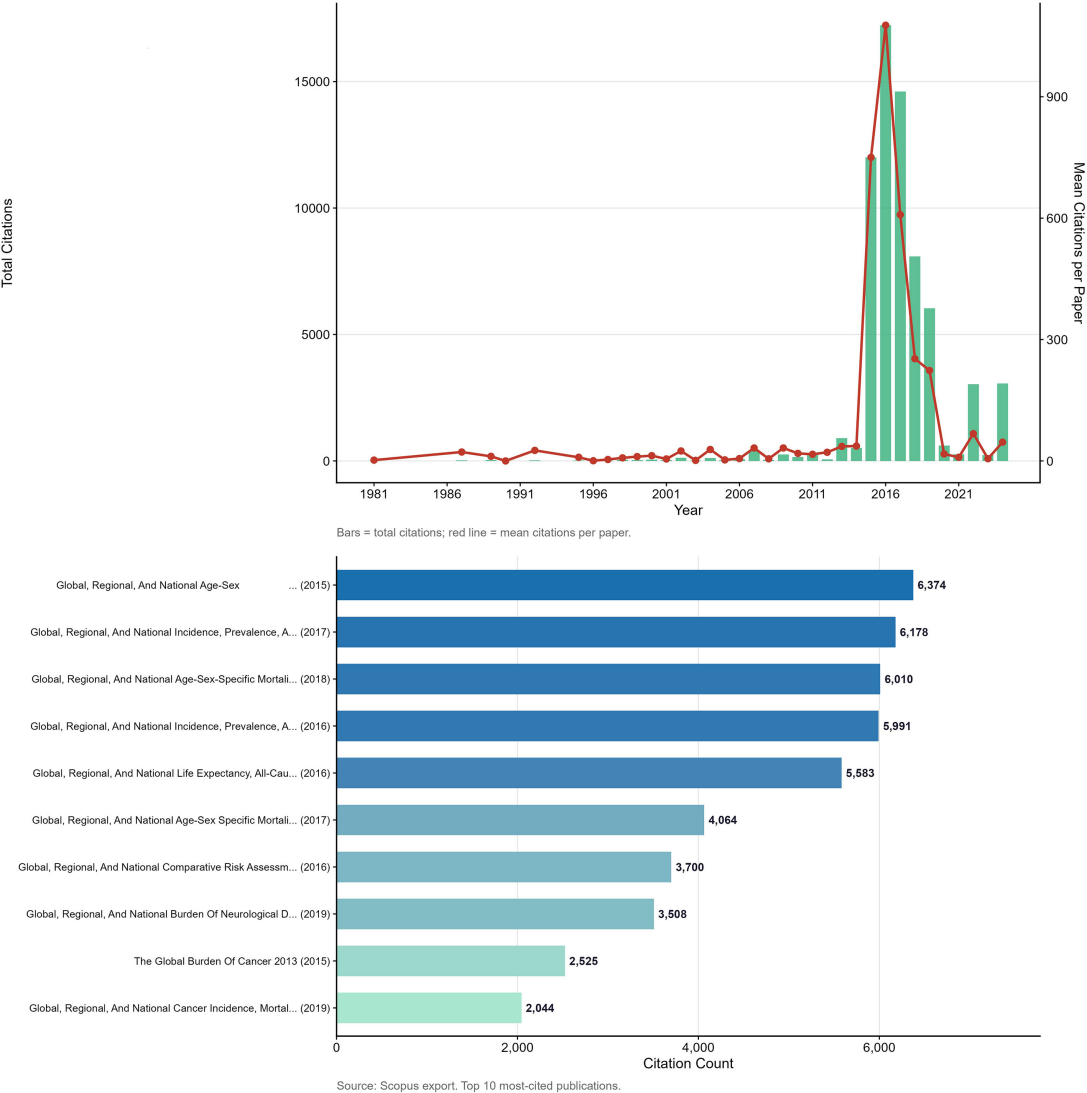
Citation trends and most-cited publications. Two-panel figure summarizing citation impact among oncology- and cancer-related publications with Bahrain affiliation or focus in the Scopus export.

The total citation count across all 502 publications was significant, with notable peaks during the 2014–2018 period, primarily driven by a select group of highly cited Global Burden of Disease (GBD) studies. The ten most-cited publications comprised large-scale multinational analyses, with citation counts ranging from 2,044 to 6,374. These publications included various iterations of the GBD cancer estimates, as well as global incidence and mortality reports. The mean number of citations per paper peaked sharply around 2015–2016, followed by a decline, aligning with the publication lag characteristic of citation accumulation for more recent papers (**Figure 6**, **Table 1**).

### 3.5 Geographic scope

**Figure 7:**
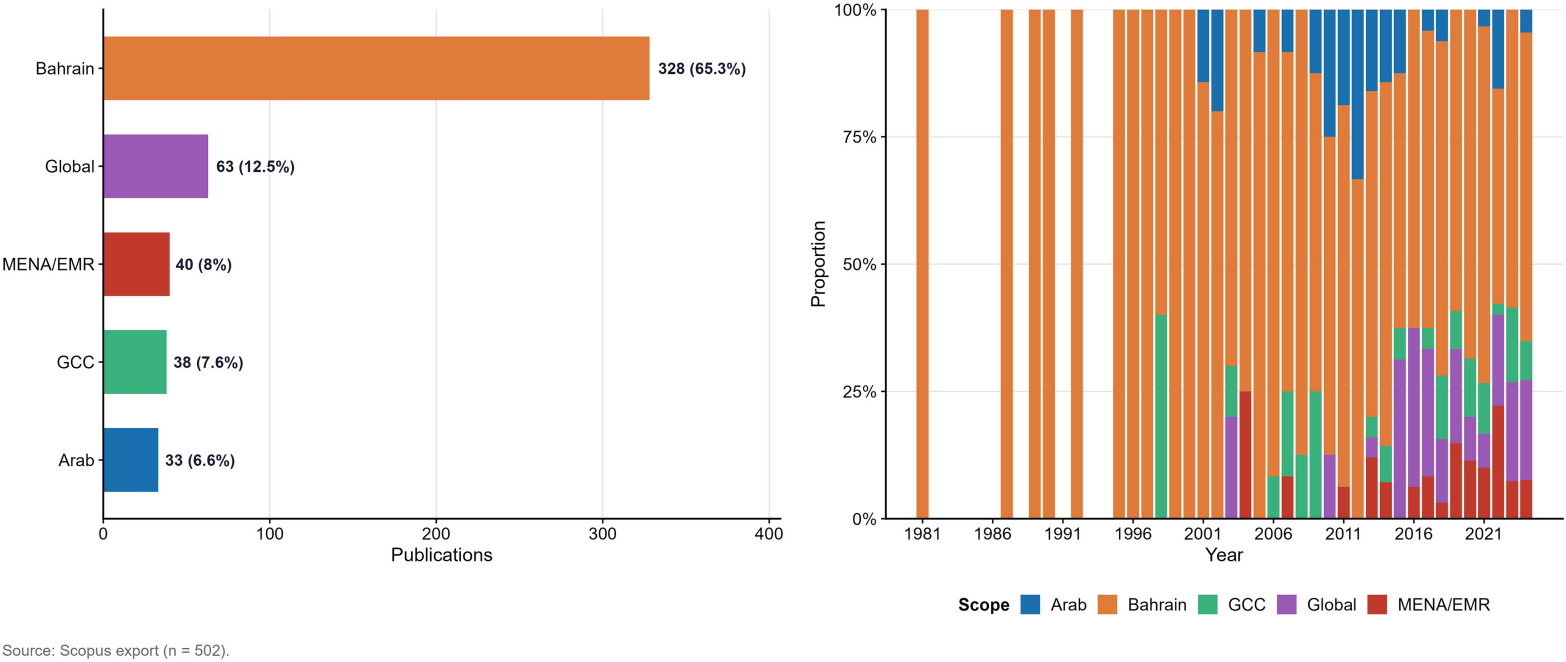
Research scope of publications. Two-panel figure summarizing the geographic research scope of oncology- and cancer-related publications with Bahrain affiliation or focus in the Scopus export.

Most publications (n=328, 65.3%) were conducted exclusively within Bahrain. Global collaborative studies accounted for 63 publications (12.5%), followed by studies at the MENA/EMR level (n=40, 8.0%), the GCC level (n=38, 7.6%), and studies from several Arab countries, including Bahrain (n=33, 6.6%). A temporal analysis of scope proportions indicated a gradual decline in exclusively Bahrain-based research, which constituted 100% of output in the earliest period, decreasing to approximately 57% by 2021–2024. Publications resulting from global collaborations, virtually nonexistent prior to 2000, accounted for approximately 17% of the annual output in the most recent period (**Figure 7**, **Table 1**).

### 3.6 Bahraini authorship and institutional contributions

**Figure 8:**
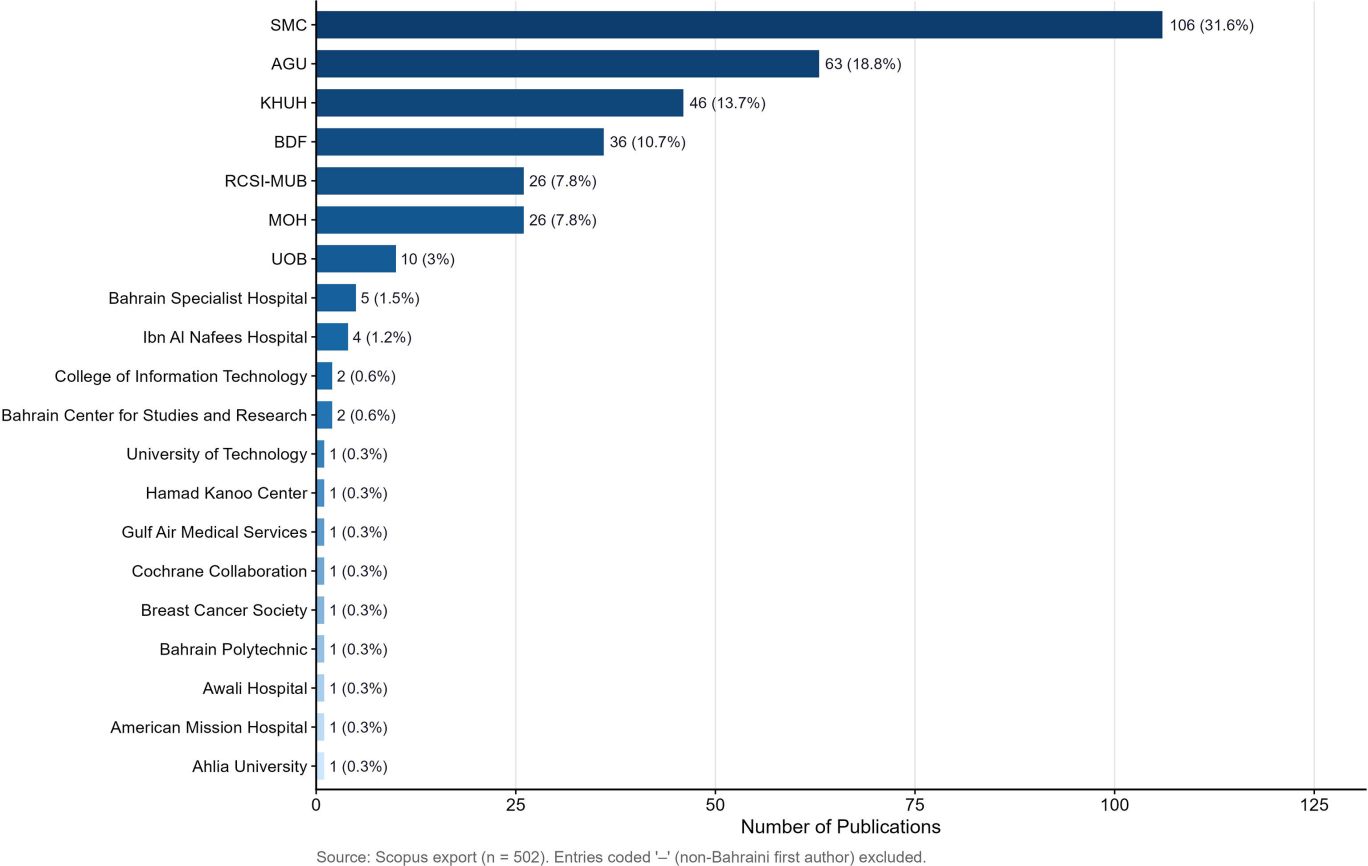
Institutional contributions to publications. Horizontal bar chart showing the leading Bahrain-based institutional affiliations among oncology- and cancer-related publications in the Scopus export.

There were 400 publications (79.7%) with at least one author from Bahrain and 335 (66.7%) with the first author from Bahrain. There was a decline in the percentage of first authors from Bahrain (p < 0.001), with the highest percentage from 1991 to 2000 (100.0%) and the lowest percentage from 2021 to 2024 (56.0%). Similarly, there was a decrease in the proportion with at least one author from Bahrain (p=0.014). (**Table 1**). Two hundred twenty-seven (67.8%) of the first authors from Bahrain were affiliated with a health institution, mainly the Salmaniya Medical Complex (SMC), the country’s largest general hospital, which had the highest number of publications (n=106, 31.6%) and accounted for almost half (46.7%) of those of health institutions. Academic institutions contributed 31.0%, with Arabian Gulf University (AGU) accounting for 60.6% of these institutions and ranking second in the country (n=63, 18.8%). King Hamad University Hospital (KHUH) generated 46 publications (13.7%), whereas Bahrain Defence Force Hospital (BDF) accounted for 36 publications (10.7%). The Royal College of Surgeons of Ireland – Medical University of Bahrain (RCSI-MUB) and the Ministry of Health (MOH) each contributed 26 publications (7.8% of the total). The University of Bahrain (UoB) contributed 10 publications (3.0%). The remaining institutions each contributed fewer than 6 publications, collectively representing less than 4% of the total output (**Figure 8**).

### 3.7 Study design

**Figure 9:**
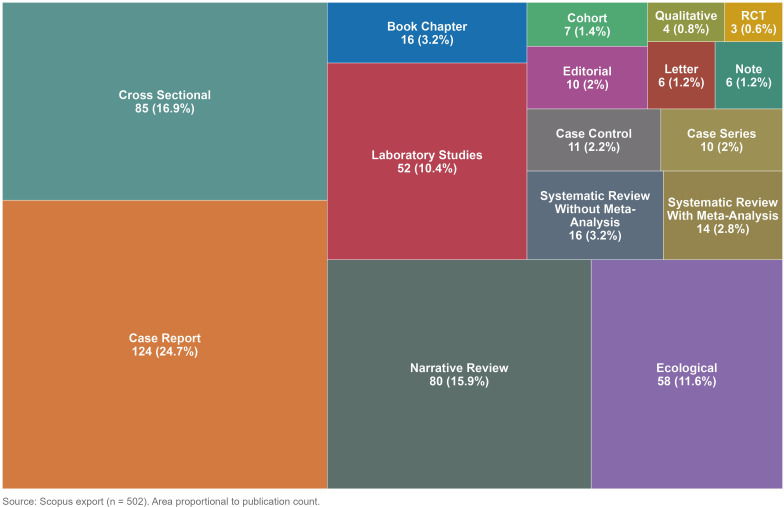
Distribution of publications by study design. Treemap depicting the distribution of oncology- and cancer-related publications with Bahrain affiliation or focus by study design, with tile area proportional to publication count.

**Figure 9** shows the study designs used in the publications. The majority were descriptive studies (n=277, 57%), followed by reviews (n=110, 22.6%), with case reports and narrative reviews accounting for 42%. There were slight changes in the study design used over the study period, with a decline in the proportion of descriptive studies (**Table 1**).

### 3.8 Cancer type

**Figure 10:**
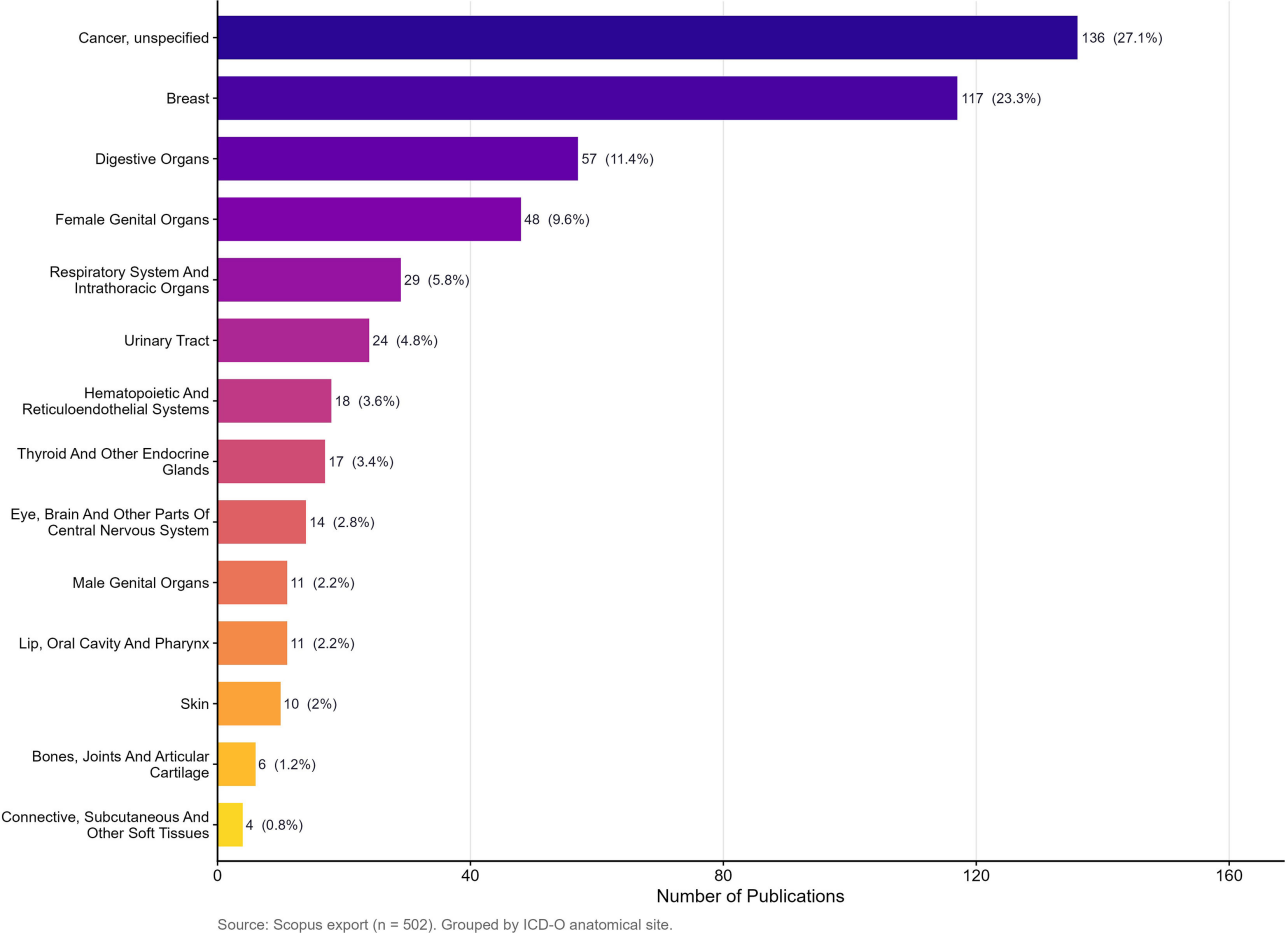
Horizontal bar chart depicting the distribution of oncology- and cancer-related publications with Bahrain affiliation or focus by cancer site, grouped according to ICD-O anatomical categories.

**Figure 10** shows the distribution of cancer types by group, and **Table 4** presents the top 10 most common cancers. “Unspecified cancer” ranked first, followed by breast cancer, together accounting for 50.4% of the publications. Colorectal cancer (5.4%) was the third most common cancer, followed by cervical cancer (4.8%), lung cancer (3.4%), thyroid and kidney cancer (3.2% and 3.2%, respectively), brain CNS cancer (2.8%), and ovarian cancer (2.6%).

**Table 4:** The top 10 most common cancers in the publications.

Rank	Cancer	Frequency	Percent
1	Cancer, unspecified	136	27.1
2	Breast	117	23.3
3	Colorectum	27	5.4
4	Cervix uteri	24	4.8
5	Lung	17	3.4
6	Thyroid	16	3.2
6	Kidney	16	3.2
8	Brain CNS	14	2.8
9	Ovary	13	2.6
10	Liver	12	2.4

**Figure 11:**
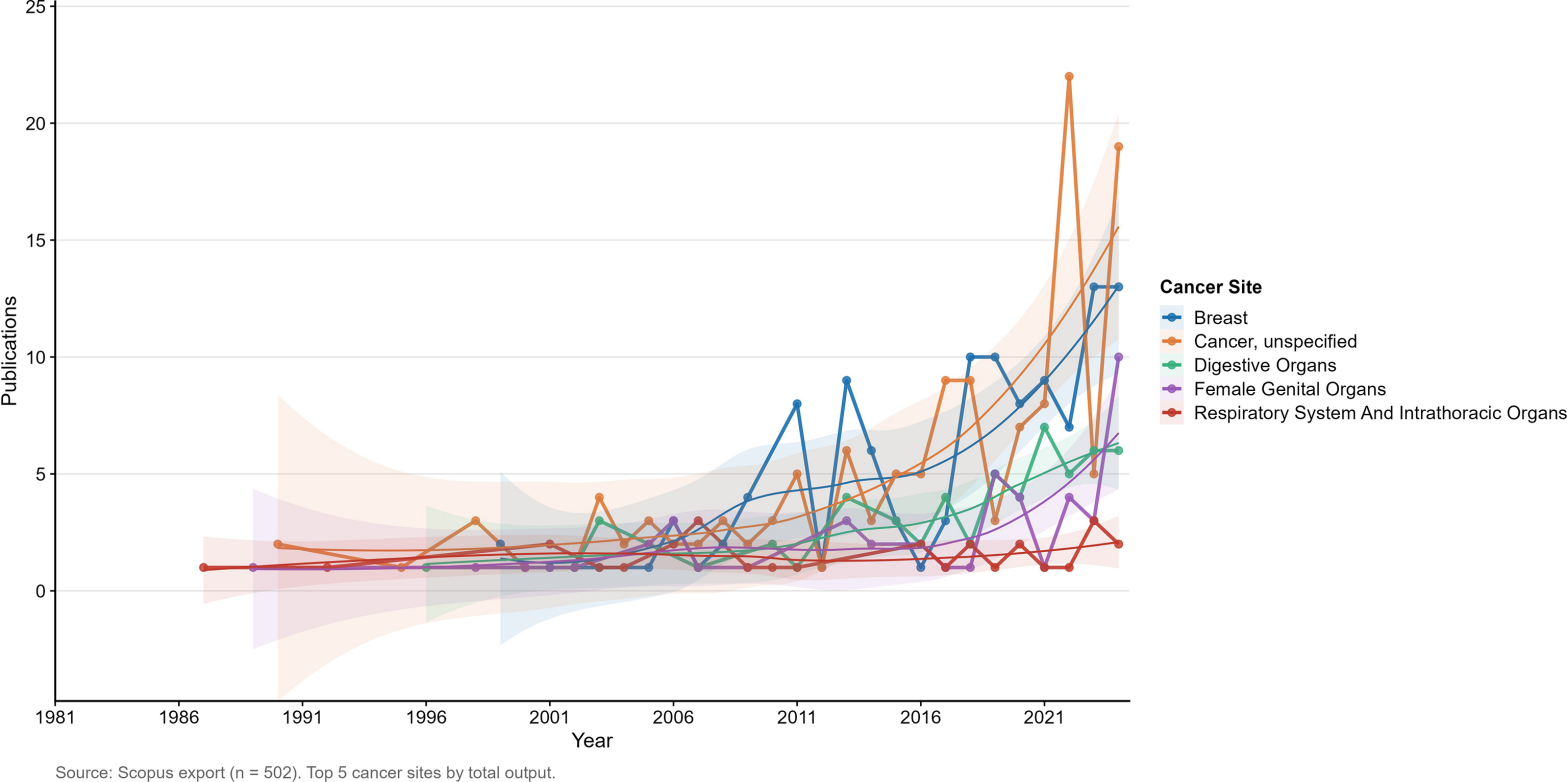
Temporal trends in publications by cancer site (1981–2024). Annual number of publications by cancer site from 1981 to 2024, showing trends for the top five sites by total output: breast cancer, digestive organs, female genital organs, respiratory system and intrathoracic organs, and unspecified cancer sites.

Among the five most extensively researched cancer site groups, “unspecified cancer” and “breast cancer” exhibited the most pronounced upward trajectories, with breast cancer demonstrating consistent growth starting in approximately 2005 and achieving its peak annual output from 2023 to 2024. Cancers of the digestive organs displayed a gradual upward trend beginning in 2010. Conversely, publications related to female genital organ cancers remained relatively rare and stable throughout most of the study period, with a modest increase observed after 2015. Research on respiratory system and intrathoracic organ cancers has remained consistently flat across all four decades, highlighting a significant gap regarding the clinical burden of this category (**Figure 11**).

A cross-tabulation of cancer sites by study design category indicated that descriptive designs predominated across nearly all cancer site groups. Breast cancer exhibited the highest absolute number of descriptive studies (n=56), followed by “unspecified cancer” (n=61) and digestive organ cancers (n=40). Analytical studies were infrequent across all sites, with the highest counts for “unspecified cancers” (n=3), breast cancer (n=5), urinary tract cancers (n=3), and female genital organs (n=4). No analytical studies were identified for respiratory system cancers, connective tissue cancers, or skin cancers. Reviews were most prevalent for breast cancer (n=23) and unspecified cancers (n=36).

## 4 Discussion

The present bibliometric analysis reveals a striking four-decade evolution in oncology research productivity in the Kingdom of Bahrain. The first publication appeared in 1981, yet output remained minimal for nearly three decades, with very low annual volumes through the early 2000s. A dramatic acceleration began approximately 2010: 77.9% of the 502 identified publications were produced between 2011 and 2024, culminating in a record of 66 papers in 2024 alone.

This surge positions Bahrain as an increasingly visible contributor to cancer research in the Arab world, marking a clear departure from its historically modest role in the region (13, 14, 17). Compared with global trends in PubMed-indexed cancer and oncology publications—which have exhibited steady, progressive growth in recent decades driven by sustained international research interest and expansion of the biomedical literature Bahrain’s trajectory stands out for its sharpness and speed. While global output has advanced consistently and moderately, Bahrain-affiliated publications shifted from prolonged low productivity to a rapid increase after 2010, with most documents appearing in the past 14 years and prominent peaks in the last year.

The observed surge in research output appears timely and responsive to the national cancer burden documented by the MOH (6, 7) and projected by recent GCC-wide estimates (4). Breast cancer accounts for both the national incidence (23.4%) and the research output (23.3%), confirming that local investigators have prioritized this leading malignancy (14). The high research output on breast cancer is not surprising, as Bahrain has the highest proportion of articles among Arab countries (14).

Similarly, colorectal cancer, which is among the top five cancers in both sexes in Bahrain, was the third most researched cancer (5.4%). However, several high-burden cancers in Bahrain—most notably lung cancer, prostate cancer, NHL, bladder cancer, and leukemia—remain underrepresented relative to their clinical importance, indicating persistent research gaps that future studies should address (5–7). A noticeable discrepancy exists between the clinical burden of lung cancer and its research prioritization in Bahrain. While lung cancer remains the second leading cause of cancer mortality in the Kingdom, it ranks fifth in terms of research frequency. This gap may be attributed to several systemic factors, including the inherent clinical complexity and higher mortality of patients with lung cancer, which often leaves a narrower window for longitudinal patient follow-up than “surviving-heavy” malignancies such as breast cancer. The relative scarcity of lung cancer studies suggests a need for increased investment in specialized local diagnostic infrastructure and targeted research funding. Furthermore, with the deliberate inclusion of “sarcoma” in the keywords and the systematic search strategy to maximize search sensitivity and ensure comprehensive capture of all malignant neoplasms in Bahrain, research focusing on “connective, subcutaneous and other soft tissues” (C49) emerged as one of the least represented categories, with only four documents (1.9%) identified. This discrepancy confirms that although the search parameters were sufficiently robust to capture these rare malignancies, the resulting low publication volume reflects a genuine and persistent research gap in the local oncology landscape and highlights sarcomas as a priority area for future research. Moreover, the proportion of publications (27.1%) that did not focus on a specific cancer was similar to that reported in a study from Botswana (27%) (19). Despite the maturation of the research landscape, the proportion of “unspecified” cancer research remained stable at approximately 25.5% from 2011 to 2020 and increased to 29.7% in the most recent period.

The continued dominance of general oncology themes in the modern era, even as publications in Q1 and Q2 journals have risen, suggests a persistent gap in granular, site-specific epidemiological and diagnostic focus. While some of these publications may represent broad policy reviews or multicancer molecular studies, the failure of this percentage to decline highlights a critical need for more targeted research into specific tumor sites to better inform site-specific clinical pathways and national screening programs. The cancer site trend analysis introduces temporal granularity to the cross-sectional distribution findings. The pronounced and sustained increase in breast cancer research since 2005 reflects the clinical and research prioritization of this predominant malignancy. In contrast, the persistent stagnation of research trajectories for respiratory system and intrathoracic organ cancers over four decades represents the most significant misalignment between cancer burden and research. Although the trajectory for digestive organ cancer research has improved somewhat, it remains modest in comparison to the status of colorectal cancer, which is the second most common malignancy in the country. These site-specific trend data should guide the prioritization framework of any national oncology research agenda.

Nearly 80% of the publications included at least one Bahraini author, and 66.7% listed a Bahraini first author, demonstrating sustained local leadership. These proportions are higher than those reported in other studies (54% and 53%, respectively) (19) and the reported percentage (57.5%) of first authors from Bahrain in medical and biomedical research in Bahrain from 2005 to 2024 (20).The majority (65.3%) of the studies were conducted exclusively in Bahrain, whereas the remainder involved international, Arab, and GCC collaboration.

The growth in overall publication volume has been accompanied by significant diversification of output types. The increasing proportion of review articles since 2010 reflects a field that is beginning to synthesize its accumulated evidence base as a hallmark of research maturation. The emergence of book chapters in the most recent period, particularly in the context of the publication of regional oncology textbooks, demonstrates that Bahraini researchers not only are producers of primary evidence but are also increasingly contributing to the regional knowledge infrastructure. The proportions of articles (71.8%) and reviews (22.5%) after excluding book chapters and conference proceedings were lower than those reported (80.2% and 12.8%, respectively) in the recent Bahraini publications on all biomedical and medical publications, most likely because of the longer period covered in our study (20). Nonetheless, the predominance of descriptive designs (57%), particularly case reports (25.5%), and the relative scarcity of analytic studies (only 4.3% of which were randomized controlled trials or cohort studies) underscore the need for capacity building in advanced research methodologies (9). This is further highlighted by an earlier study, which also revealed that descriptive studies accounted for 45.9%, case reports for 32.4%, and clinical trials were not reported (13). The scarcity of clinical trials across all periods might be due to inequalities in cancer research in sponsored clinical trials (21) and highlights a gap that future capacity-building initiatives should specifically address.

The two top journals, Bahrain Medical Bulletin and the Journal of Bahrain Medical Society, are local medical journals that account for 25% of all publications. This is similar to what was reported for medical and biomedical research productivity in Bahrain, where these two journals ranked first and second, accounting for 50.5% of the total (20). Furthermore, Bahrain Medical Bulletin ranked 15th among the top 20 cancer journals in Arab countries (14). A bibliometric cancer study from Iran revealed that although the top journal was the Asian Pacific Journal of Cancer Prevention (5.0%), 13 Iranian journals (10.4%) were among the top 18 (22). The prominent role of the Bahrain Medical Bulletin underscores the vital importance of local journals in documenting clinical observations and specific epidemiological data at the local and regional levels. However, the historical dominance of this local venue, which frequently publishes a high volume of case reports, significantly influences the overall bibliometric profile of Bahraini research, as it typically receives fewer citations. Furthermore, these publications have limited international dissemination compared with major global databases. This context provides a necessary perspective on the strategic shift observed after 2010, as Bahraini researchers increasingly targeted Q1 and Q2 international journals. The journal SJR by decade supports and expands upon the quartile findings. The transition from the narrow, low-value SJR distributions observed in the 1990s and 2000s to the significantly wider and higher distributions in the 2010–2019 and 2020–2024 periods indicates a substantial qualitative shift in publication venues targeted by Bahraini oncology researchers. The bimodal nature of the 2010–2019 distribution, characterized by a large cluster of low-SJR publications alongside a tail of very high-SJR multinational collaborative papers, highlights the dual character of contemporary research output: a growing core of locally driven studies published in international yet mid-tier journals, alongside a smaller number of prestigious collaborative publications. This transition reflects a maturing research ecosystem moving toward higher-impact venues to enhance the visibility, citation potential, and global integration of the Kingdom’s scientific contributions. These findings align with bibliometric patterns observed in other studies from Bahrain (20) and in emerging research fields (10) and suggest that Bahraini oncology researchers are increasingly targeting higher-impact outlets. To sustain and enhance this trend, continued mentorship, grant support, and institutional incentives that target higher-impact venues are essential. The citation analysis reveals a critical nuance in interpreting Bahrain’s bibliometric profile. The significant citation peaks observed during the 2014–2018 period are predominantly attributable to a limited number of large multinational collaborative publications, notably the GBD studies, in which Bahraini authorship reflects participation in extensive international networks rather than the generation of primary data within the country. While inclusion in such high-profile publications markedly enhances aggregate citation metrics and indicates Bahrain’s integration into global research communities, it also implies that the citation record does not accurately reflect the local generation of original evidence. This distinction is vital for policymakers: the high citation impact of collaborative papers should not replace the necessity of investing in locally driven, hypothesis-generating research that directly addresses the cancer burden in Bahrain.

The institutional analysis highlights a research landscape characterized by a limited number of institutions, with SMC and AGU collectively representing over half (50.4%) of all first-author publications from Bahrain. This concentration is understandable, as SMC is the nation’s largest tertiary referral center and a main hub for oncology clinical activity, whereas AGU serves as the leading academic medical institution. However, the emergence of KHUH (13.7%) and RCSI-Bahrain (7.8%) as significant contributors indicates a positive expansion of the institutional base. Notably, the MOH’s contribution as an authoring institution (7.8%) is particularly significant, reflecting the active involvement of public health authorities in generating cancer research evidence rather than merely consuming it. However, the limited contributions from private hospitals and the near absence of basic science research institutions reveal structural gaps in Bahrain’s oncology research infrastructure that could benefit from targeted investment and enhanced interinstitutional collaboration frameworks.

The growing internationalization of Bahraini cancer research merits particular attention. The proportion of studies conducted exclusively within Bahrain declined from 100% in the 1980s to 57.1% from 2021 to 2024, whereas the proportion of collaborative publications rose from 0% to 17.0% globally over the same period. This shift toward multicountry partnerships is a hallmark of maturing national research ecosystems and is consistent with patterns observed across the GCC countries and Arab-world bibliometric studies (13–15). Furthermore, the observed shift in authorship patterns, specifically the decline in Bahraini first authorship from 100% in 1991–2000 to 56% in 2021–2024, represents a significant evolutionary milestone in the Kingdom’s research trajectory. Rather than indicating a reduction in local research initiative, this trend serves as a hallmark of “internationalization.” Bahrain’s small population and relatively compact healthcare system may paradoxically be an asset in this regard, enabling efficient national data linkages and positioning the country as an attractive partner in regional multicenter trials. Sustaining and expanding these collaborative networks, particularly with GCC neighbors and global cancer consortia, should therefore be a strategic priority for national research funding bodies. While lead authorship in such large-scale collaborations often falls to primary coordinating centers, the inclusion of Bahrain-affiliated researchers in these prestigious networks signifies that the country is now a recognized partner in the global oncology community.

Bahrain’s chronically low research and development expenditure, reported at only 0.19% of GDP in 2023, represents a structural constraint that likely underlies many of the methodological shortcomings identified in this study (13, 14). The preponderance of descriptive studies and case reports, the relative scarcity of randomized controlled trials, and the underinvestment in basic and translational science are consistent consequences of limited dedicated funding for oncology research. In contrast, countries that have committed greater proportions of their GDP to R&D, such as the United Arab Emirates (1.49%) and Qatar (0.68%), have demonstrated more diversified research portfolios and higher rates of internationally indexed output (13, 14, 23). Closing this funding gap will require coordinated action across multiple sectors that jointly develop a national oncology research agenda with ring-fenced funding streams, competitive grant mechanisms, and protected research time for clinical investigators and researchers. This study provides a novel and timely contribution as the first long-term bibliometric analysis of oncology research in Bahrain, offering a comprehensive dataset spanning four decades (1981–2024) and filling a clear gap in both the national literature and the Arab-world literature. A key strength lies in its policy-oriented approach, which links academic output directly to institutional planning and regional clinical burdens. The methodology was rigorous and transparent: publications were identified through systematic searches of electronic databases, supplemented by manual verification, duplicate removal, and explicit inclusion/exclusion criteria, resulting in a comprehensive dataset of 502 documents. Detailed stratification by temporal trends, authorship patterns (local first author and at least one author from Bahrain), geographic scope, study design, journal quality (quartiles and SJR), citation impact, and specific cancer types provides granular, actionable insights rarely available in similar regional bibliometric studies. Finally, the findings are directly contextualized against Bahrain’s national cancer incidence and mortality data, offering policymakers and funding bodies an evidence-based roadmap for strategic research investment and capacity building.

However, the study has several limitations that must be acknowledged. First, while the study utilized a rigorous systematic search across Scopus and PubMed/MEDLINE and adhered to the PRISMA 2020 reporting principles for transparency in data selection, the scope was restricted to these two major databases. This may introduce a degree of selection bias by omitting gray literature or regional journals not indexed in these repositories. Second, the analysis is primarily descriptive and focused on performance indicators such as publication counts and journal impact tiers. It does not incorporate advanced science mapping techniques such as co-authorship networks or keyword co-occurrence analysis—which could offer deeper inferential insights into the field’s intellectual and social structures. Third, because this is a single-country study, the findings are highly context-specific to Bahrain’s healthcare and academic infrastructure; therefore, while the results offer a valuable blueprint for other GCC and Arab nations, they can be generalized with caution to broader international settings. Collectively, these findings provide a foundational evidence base for strategic oncology planning in Bahrain and the broader GCC region. The transition from local case reporting to high-impact international collaboration highlights a maturing research ecosystem; however, the persistent gaps identified, specifically the disconnect between high-mortality sites such as lung cancer and their corresponding research outputs, underscore the need for policy-driven resource allocation. To sustain this momentum, institutional stakeholders should prioritize funding for site-specific epidemiological studies and provide structural support for local investigators to move into senior authorship roles within global networks. By aligning research priorities with national cancer registry data and clinical burden. Bahrain can further refine its transition from descriptive oncology to high-value, translational research that directly informs regional screening programs and therapeutic protocols.

While the present study provides a robust performance analysis and a clinically structured thematic overview using ICD-O classifications, it did not employ automated science mapping techniques such as keyword co-occurrence or co-authorship network analysis. Our approach prioritized a longitudinal, classification-based analysis to ensure clinical relevance for local health policy and to establish a reproducible baseline. Future research utilizing bibliometric software such as VOSviewer or Bibliometrix could build upon this foundation by mapping the specific clusters of research topics and the evolving collaborative nodes between Bahraini and international institutions, providing a deeper view of the intellectual and social structures driving the region’s scientific output.

A further limitation concerns the absence of normalization in the primary bibliometric analysis. Publication counts are presented in absolute terms and are not adjusted for population size, gross domestic product (GDP), or the number of active researchers in Bahrain over the study period. Although per-capita comparisons are discussed to provide contextual benchmarking, the core analysis remains descriptive in absolute terms. This constrains the direct comparability of findings across countries or time periods that differ substantially in population scale or economic capacity, and should be considered when interpreting cross-national performance differences.

## 5 Conclusions

This analysis addresses a critical gap in the literature by providing the first comprehensive, long-term bibliometric profile of oncology research in Bahrain. The findings offer actionable insights for policymakers: sustained investment in analytic and interventional research, targeted funding for understudied malignancies, and a continued emphasis on local authorship within international collaborations will be essential for translating research growth into measurable improvements in cancer outcomes. Bahrain’s trajectory demonstrates that strategic national commitment can rapidly increase research productivity in a small country and serve as a model for other GCC and Arab nations facing similar epidemiological transitions.

## Data Availability

The original contributions presented in the study are included in the article/supplementary material. Further inquiries can be directed to the corresponding author.

## References

[1] Force LM Kocarnik JM May ML Bhangdia K Crist A Penberthy L . The global, regional, and national burden of cancer, 1990–2023, with forecasts to 2050: a systematic analysis for the Global Burden of Disease Study 2023. Lancet. (2025) 406:1565–86. doi: 10.1016/s0140-6736(25)01635-6 41015051 PMC12687902

[2] Luo Q Smith DP . Global cancer burden: progress, projections, and challenges. Lancet. (2025) 406:1536–7. doi: 10.1016/s0140-6736(25)01570-3 41015053

[3] Siegel RL Kratzer TB Giaquinto AN Sung H Jemal A . Cancer statistics, 2025. CA Cancer J Clin. (2025) 75:10–45. doi: 10.3322/caac.21871 39817679 PMC11745215

[4] Alessy SA Alqahtani SA Vignat J Abuhmaidan A Basmi AEL Al Lawati N . The current and future cancer burden in the Gulf Cooperation Council (GCC) countries. Cancer Med. (2024) 13:e70141. doi: 10.1002/cam4.70141 39279725 PMC11403302

[5] Ferlay J Ervik M Lam F Laversanne M Colombet M Mery L . Global Cancer Observatory: Cancer Today. Lyon, France: International Agency for Research on Cancer (2024). Available online at: https://gco.iarc.who.int/today (Accessed January 1, 2026).

[6] Ministry of Health . Cancer incidence & mortality in the Kingdom of Bahrain-1998-2016-Statistics and trends 2016. Manama, Bahrain: Ministry of Health (2017).

[7] Ministry of Health . 10 years 2009-2018 Cancer incidence & mortality in the Kingdom of Bahrain-Statistics and trends. (2022).

[8] WHO . The WHO strategy on research for health. Geneva: WHO (2012). Available online at: https://iris.who.int/server/api/core/bitstreams/642b0fea-9205-4a35-bc06-dfc9b3b7ad13/content (Accessed January 1, 2026).

[9] Are C Yanala U Malhotra G Hall B Smith L Cummings C . Global curriculum in research literacy for the surgical oncologist. Eur J Surg Oncol. (2018) 44(1):31–42. doi: 10.1016/j.ejso.2017.07.017 29242017

[10] Iweka E Ezenwuba BN Snaith B . A bibliometric analysis on research authorship and collaboration patterns in radiography professional journals: A 10-year review. J Med Imaging Radiat Sci. (2025) 56:101772. doi: 10.1016/j.jmir.2024.101772 39504642

[11] Pugh K Gyllingberg L Stratiev S Hamis S . A bibliometric study on mathematical oncology: interdisciplinarity, internationality, collaboration and trending topics. Bull Math Biol. (2025) 87:174. doi: 10.1007/s11538-025-01544-9 41184520 PMC12583306

[12] Cui J Xiong Y Sun M Gu X Liu Y Zhong L . Trends in immune-related adverse events for colorectal cancer: A bibliometric analysis. Front Oncol. (2022) 12:1024321. doi: 10.3389/fonc.2022.1024321 36387099 PMC9646945

[13] Hamadeh RR Borgan SM Sibai AM . Cancer Research in the Arab World: A review of publications from seven countries between 2000-2013. Sultan Qaboos Univ Med J. (2017) 17:e147–54. doi: 10.18295/squmj.2016.17.02.003 28690885 PMC5488814

[14] Hamadeh RR Jahrami H Nazzal K . Cancer research in the Arab World. In: Al-Shamsi HO Abu-Gheida IH Iqbal F Al-Awadhi A , editors.Cancer in the Arab World. Springer, Singapore (2022). p. 395–408.

[15] Alhomoud S Al-Othman S Al-Madouj A Homsi MA AlSaleh K Balaraj K . Progress and remaining challenges for cancer control in the Gulf Cooperation Council. Lancet Oncol. (2022) 23:e493–501. doi: 10.1016/s1470-2045(22)00488-0 36328023

[16] Browser UD . GERD as percenatage of GDP United States. UNESCO (2026). Available online at: https://databrowser.uis.unesco.org/ (Accessed January 1, 2026).

[17] Hamadeh RR Jahrami H . Cancer care in Bahrain: progress, challenges, and strategic priorities. Lancet Oncol. (2025) 26:830–1. doi: 10.1016/s1470-2045(25)00226-8 40516556

[18] Organization WH . International Classification of Diseases for Oncology (ICD-O). Geneva, Switzerland: The World Health Organization (WHO) (2013).

[19] Koobotse MO Zachariah M Senabye B Gobe I Kadimo K Nthontho KC . Bibliometric analysis of cancer research outputs in Botswana between 2009 and 2021. J Cancer Policy. (2023) 35:100405. doi: 10.1016/j.jcpo.2023.100405 36690157 PMC10066854

[20] Hamadeh RR Khedr E Jahrami H . Medical and biomedical research productivity in Bahrain: an analysis of gender differences and patterns over two decades. Front Res Metr Anal. (2026) 11:1714436. doi: 10.3389/frma.2026.1714436 41717261 PMC12913561

[21] Payedimarri AB Mouhssine S Aljadeeah S Nkubito BM Gaidano G Ravinetto R . Assessing patterns of authorship of low- and middle-income countries in global commercial clinical trials in oncology. Global Health. (2025) 22:3. doi: 10.1186/s12992-025-01167-8 41275285 PMC12763880

[22] CheshmehSohrabi M Shabani R Shirdavani S . Tops and trends in Iranian cancer research: A bibliometric analysis. Arch Iran Med. (2022) 25:224–34. doi: 10.34172/aim.2022.38 35942994 PMC11897874

[23] Pedapenki R Madan A . General oncology care in Bahrain. In: Al-Shamsi H Abu-Gheida I Iqbal F Al-Awadhi A , editors.Cancer in the Arab World. Springer, Singapore (2022). p. 31–6.

